# Optical Coherence Tomography Angiography in Glaucoma

**DOI:** 10.4274/tjo.53179

**Published:** 2018-09-04

**Authors:** Gábor Holló

**Affiliations:** 1 Semmelweis University, Department of Ophthalmology, Unit of Head, Glaucoma and Perimetry, Budapest, Hungary

**Keywords:** Glaucoma, OCTA, optical coherence tomography angiography, perfusion, peripapillary retinal nerve fiber layer, macula

## Abstract

Optical coherence tomography angiography (OCTA) comprises different OCT-based technologies developed for non-invasive assessment and measurement of optic nerve head and retinal perfusion. Currently the most clinically established approach is based on the split spectrum amplitude decorrelation algorithm, which detects moving red blood cells and eliminates all other information. The two main clinical fields in which OCTA offers clinically useful information are investigation of the macular retina (e.g. in macular degeneration and diabetic macular disease) and glaucoma. For glaucoma, the optic nerve head and the peripapillary retinal perfusion in the retinal nerve fiber layer, and the superficial perifoveal macular vasculature are the areas of interest. This review provides a comprehensive summary of the most important current and potential future applications of OCTA in glaucoma, but it does not address the nonglaucomatous optic nerve head or peripapillary and macular diseases.

## What is Optical Coherence Tomography Angiography?

Optical coherence tomography angiography (OCTA) comprises various OCT-based technologies.^1^ These methods all offer noninvasive assessment of perfusion in various retinal layers separately, or allow assessment of iris and corneal neovascularization and filtering bleb vascularity. Various instrument families from different manufacturers (e.g. Optovue, Zeiss, Heidelberg, Topcon, Nidek and Canon) are currently used in clinical practice. All of these systems offer perfusion images but currently not all instrument and software versions provide objective measurements. It is important to note that due to the technical differences, conversion of the parameters and measured values between the different instrument systems is not possible.

**Figure 1:**
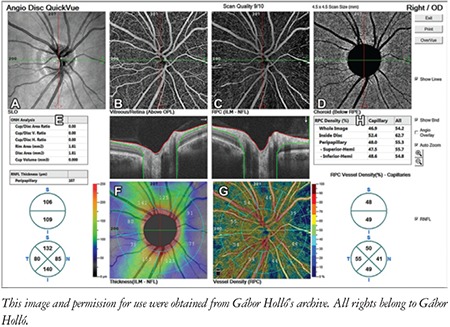
Peripapillary optical coherence tomography angiography (OCTA) report of a healthy eye with the Optovue OCTA system. A) Scanning laser ophthalmoscopy image of the measurement area; B) Vitreous-retinal perfusion map. C) Perfusion map in the radial peripapillary capillaries layer which corresponds to the retinal nerve fiber layer. D) Perfusion map in the choroid level. E) Optic nerve head and retinal nerve fiber layer thickness measurement results. F) Color-coded retinal nerve fiber layer thickness image. G) Color-coded vessel density map and its sectors in the radial peripapillary capillaries layer. H) Capillary and all-vessels density values for various sectors of the peripapillary area in the radial peripapillary capillaries layer RNFL: Retinal nerve fiber layer, OD: Right eye, ILM: Internal limiting membrane, NFL: Nerve fiber layer

**Figure 2:**
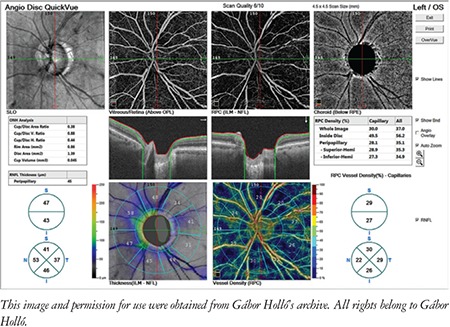
Peripapillary optical coherence tomography angiography report of an eye with advanced glaucomatous damage RNFL: Retinal nerve fiber layer, OS: Left eye, ILM: Internal limiting membrane

**Figure 3:**
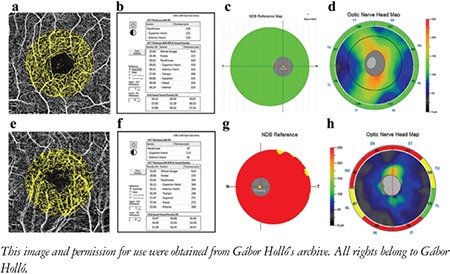
Superficial perifoveal macular vessel density and the corresponding macular inner retina thickness and retinal nerve fiber layer thickness in a healthy eye (A to D) and in an advanced glaucoma eye (E to H). A and E: Color coded perfusion maps, B and F: Vessel density measurement values, C and G: Macular inner retina thickness maps, D and H: Retinal nerve fiber layer thickness maps

This review and the presented images are based on the most commonly used OCTA system, the Angiovue OCT (Optovue Inc., Fremont, CA, USA), but the main findings are also valid for most other OCTA instruments. In the Angiovue OCT, the measurement principle is based on split spectrum amplitude decorrelation algorithm (SSADA).^1^ This algorithm detects red blood cell movement independently from the direction of movement. At the same time, all static (structural) information is removed by the software. Thus, in contrast to fluorescein angiography, data in OCTA are derived from the red blood cells rather than plasma. It is important to note that “nonperfusion” in OCTA does not necessarily mean missing or obstructed vessels, or lack of capillary plasma perfusion; it simply means that at the time of image acquisition, no moving red blood cells were present in the “nonperfused” areas. For both research purposes and glaucoma practice, the most informative parameters are peripapillary vessel density (angioflow vessel density) in the peripapillary retinal nerve fiber layer (RNFL) (Figures 1 and 2) and superficial perifoveal vessel density in the macula (Figure 3).^1^ Vessel density is the perfused area expressed as a percentage of the total examined area or its predefined sectors within a retinal layer of interest.^1,2^ OCTA of the disc area is technically available in most systems, but the presence of the large vessels and the variability and complexity of the three-dimensional optic nerve head structure make the measured results difficult to interpret. In order to correctly interpret the results, both the OCTA image and the corresponding structural image (en face OCT image) need to be used (Figures 1 and 2).^3,4^ In the most advanced systems, capillary vessel density and all-vessel density are measured separately for each retinal layer.^5,6^

## Are Optical Coherence Tomography Angiography Measurements Reliable?

Retinal perfusion is autoregulated, but vascular dysfunction is common in open-angle glaucoma.^7^ In addition, systemic blood pressure and other systemic parameters may vary between visits, as is commonly seen in the elderly glaucoma population. Therefore, short-term repeatability and long-term reproducibility of vessel density measurements in the peripapillary RNFL and the superficial perifoveal macular layer were investigated by several groups.^3,8,9^ In general, both parameter types show high repeatability and long-term reproducibility in both normal and glaucomatous eyes when the image quality is high. In our reproducibility study, the long-term reproducibility of peripapillary vessel density measurements was less than 4%,^3^ and in another study the between-visit reproducibility of superficial perifoveal macular vessel density was less than 9%.^9^ We and others have also shown that long-term reproducibility of peripapillary vessel density measurements is independent of RNFL thickness, thus the reproducibility is similar in healthy, early and advanced glaucomatous eyes.^3,8^ Another important question is whether peripapillary vessel density values reflect the perfusion of the peripapillary RNFL thickness. We and others have consistently found a strong relationship between vessel density and glaucomatous damage through the spectrum of glaucoma disease severity.^3,10,11^

## Can Vessel Density Measurements Be Used to Diagnose Glaucoma?

For some instrument and software types, differentiation of open-angle glaucoma and normal eyes using peripapillary vessel density or superficial perifoveal macular vessel density measurements is similar to of even better than that with RNFL thickness.^12^ However, the accuracy of the different instrument systems seems to vary considerably.^12,13,14,15,16,17^ Currently no head-to-head comparison study is available. Using the Angiovue OCT system, discrimination between perimetric open-angle glaucoma eyes and normal eyes was stronger with peripapillary vessel density than RNFL thickness,^12^ but the opposite was found for distinguishing angle-closure glaucoma eyes from normal eyes.^15^ Together with other results shown later in this review, this result suggests that for open-angle glaucoma, in which vascular dysregulation frequently plays a role in the development of the disease, vessel density measurements may offer advantages in early diagnosis; in contrast, in angle closure glaucoma, in which intraocular pressure elevation has a major or exclusive pathophysiological role, the structural parameters perform better.

**Figure 4:**
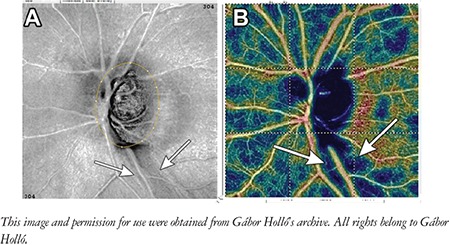
A localized retinal nerve fiber bundle defect and the spatially corresponding localized peripapillary vessel density reduction (arrows) in glaucoma

It was shown that localized RNFL bundle defects are spatially associated with localized peripapillary vessel density reductions (Figure 4), even in early and preperimetric open-angle glaucoma.^4^ Strong spatial correspondence between peripapillary vessel density reduction and deep lamina cribrosa defects was also confirmed.^18^

## Structure-function Relationship with Optical Coherence Tomography Angiography

In OCTA, the “structure-function relationship” is in fact a “function-function relationship” in which localized and generalized OCTA parameters are related to the spatially corresponding visual field sensitivity or damage values. However, the most interesting part of these investigations is when structural parameters (RNFL thickness, sector RNFL thickness, inner macular retina thickness and its hemifields) are also included in the analysis. In general, for both the Humphrey and Octopus perimetry systems a strong negative relationship was found between peripapillary, whole image, and macular vessel density parameters and the corresponding visual field deterioration.^19,20,21^ It is even more interesting that in primary open-angle glaucoma, the relationship is particularly strong for superotemporal and inferotemporal peripaillary vessel density and the spatially corresponding visual field sectors, and this relationship can be significantly stronger than that seen for the spatially corresponding sector RNFL thickness value.^19,20^ These results support the pathophysiological hypothesis which suggests a causative role of vascular dysregulation in the development and progression of primary open-angle glaucoma, particularly in the inferotemporal and superotemporal peripapillary areas, where glaucomatous RNFL loss appears early and progresses rapidly.^7^

It is also interesting that a strong relationship was found between mean paracentral visual field defect values in Octopus perimetry and temporal peripapillary vessel density values.^22^ The temporal peripapillary area (the papillomacular RNFL bundle) has been considered a particularly stable sector of the RNFL which does not thin until the latest stages of glaucoma. However, the perfusion-function relationship suggests that some mild vascular dysfunction or damage may start in the papillomacular area much earlier than previously thought. Recently a similar relationship was published for superficial perifoveal macular vessel density and mean sensitivity of the central 10-degree visual field in Humphrey perimetry,^23^ and correspondence was found between the presence of central visual field defects and increased size of the foveal avascular zone in glaucoma.^24^

## Myopia and Peripapillary Vessel Density

Since RNFL thickness measurements are influenced by myopia and atypical optic nerve head morphology, it was important to investigate the clinical applicability of vessel density measurements in myopic eyes.^25,26,27^ Few studies to date have addressed this problem. In glaucoma with high myopia, the regional relationship of visual field and peripapillary vessel density is significantly greater than that with the corresponding RNFL thickness.^25^ In myopia without glaucoma, peripapillary vessel density is lower than in normal eyes, and in myopic glaucoma it is even more reduced.^26^ Similarly to RNLF thickness, perifoveal perfusion is altered in myopia with disc torsion.^27^ Thus, in myopic glaucoma we cannot expect considerably better diagnostic accuracy from OCTA than from structural OCT parameters.

## Image Quality and Artifacts in Optical Coherence Tomography Angiography

**Figure 5:**
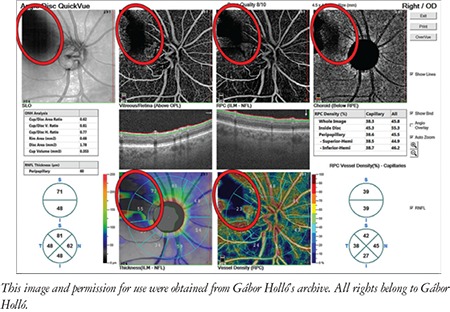
Influence of a vitreous floater (encircled with a red ellipse) on the peripapillary optical coherence tomography angiography measurement RNFL: Retinal nerve fiber layer, OD: Right eye, ILM: Internal limiting membrane, NFL: Nerve fiber layer

Similar to structural OCT measurements, OCTA is seriously influenced by image artifacts, most commonly vitreous floaters. Collagen opacities, which are common in glaucoma,^28^ may cause shadow effect by blocking the return of the illumination light from the retina.^29^ The shadow effect results in an OCT image and vessel density values which falsely imitate reduced or missing perfusion (Figure 5). Other artifacts known in structural OCT imaging may also have a significant impact on OCTA image quality and the measured values.^29,30^

## Can Optical Coherence Tomography Angiography Be Used to Distinguish Glaucoma from Other Optic Nerve Diseases?

**Figure 6:**
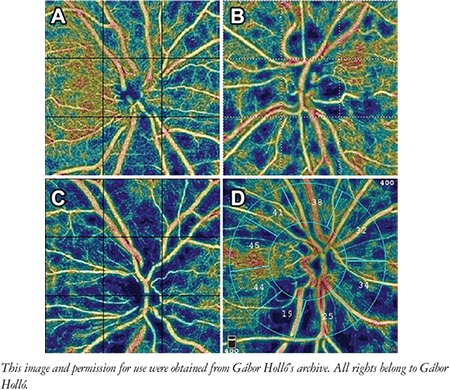
Color-coded peripapillary optical coherence tomography angiography images of four eyes with four different optic nerve head diseases. A) Compression due to optic nerve head drusen, B) Retinal vein occlusion earlier in life, C) Chronic non-arteritic anterior ischemic optic neuropathy, D) Advanced glaucoma

The peripapillary OCTA alterations are unfortunately not disease-specific. Reduction of perfusion is determined by disease severity rather than disease type.^30^ Figure 6 illustrates how different optic nerve head diseases appear very similar in OCTA images. Thus, detailed evaluation of the optic nerve head and peripapillary retina remains mandatory for disease classification even in the age of modern OCTA technology.

## Influence of Intraocular Pressure on Measured Vessel Density Values

**Figure 7:**
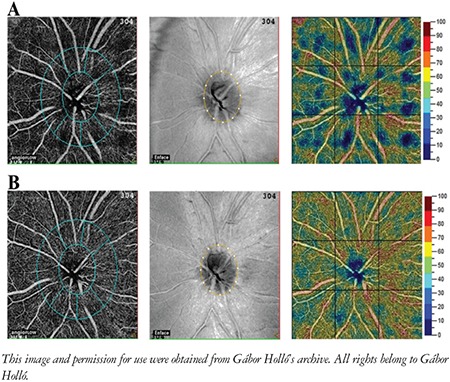
Influence of large intraocular pressure reduction on peripapillary vessel density in ocular hypertension. A) Untreated eye with high intraocular pressure. B) The same eye one month later under combined intraocular pressurelowering medication and with more than 50% intraocular pressure reduction. The peripapillary vessel density map shows a clear increase in capillary perfusion

The potential relationship between intraocular pressure or intraocular pressure reduction and peripapillary perfusion has been an important question in glaucoma management for decades. The structural parameters (RNFL thickness and inner macular retina thickness) do not react in an active way to pressure-lowering treatment. In a proof-of-concept investigation conducted on systemically healthy, young, and newly diagnosed open-angle glaucoma and ocular hypertensive patients whose untreated intraocular pressure was high but decreased by at least 50% to 18 mmHg or less under treatment, we found a clinically very significant improvement in the initially reduced peripapillary vessel density (Figure 7).^31^ Later other investigators using other OCTA systems and intraocular pressure-lowering interventions confirmed our results.^32,33^ The results achieved with intraocular pressure reduction suggest that in relatively early glaucoma, the change of vessel density may potentially be used for early assessment of the interventions’ long-term beneficial effect on visual field preservation.

## Can We Use Vessel Density for the Measurement of Glaucomatous Progression?

The current standard of detection and measurement of glaucomatous progression is serial visual field testing and software-based visual field progression analysis. However, structural OCT parameters (RNFL thickness, inner macular retina thickness) have also been investigated for their applicability for the measurement of glaucomatous progression. Recently, structural progression measurements became a part of modern glaucoma care and progression analysis. In some of our latest studies we prospectively investigated early (2-year) glaucomatous progression with peripapillary vessel density and RNFL thickness measurements.^34,35^ Progression was better detected with RNFL thickness progression analysis. Statistically significant peripapillary capillary vessel density progression was found in 17% of the study eyes, and half of the progressing cases also showed significant and spatially corresponding RNFL thickness progression.^35^ It is important to note that in contrast to structural OCT parameters, which are not under physiological regulation, vessel density reflects intraocular pressure changes, status of systemic perfusion, glaucomatous vascular dysregulation, retinal oxygenation, and hypercapnia. Thus, vessel density is less stable than RNFL thickness. Therefore, we cannot expect to see an easy-to-understand vessel density progression pattern when the gradual glaucoma-related perfusion changes are small, or smaller than the fluctuations induced by factors which are not related to glaucomatous progression.

In another recent investigation, we evaluated the potential influence of breath holding on vessel density measurements using a standardized and extreme form of breath holding, the Valsalva maneuver.^36^ Breath holding and the Valsalva maneuver do influence retinal perfusion. Due to the increased intrathoracic pressure, the venous outflow from the eye decreases and as a consequence, retinal perfusion is temporally reduced. Since vessel density measurements are time-consuming (image acquisition for peripapillary measurements may take up to 16 seconds), the reduction of capillary perfusion in the RNFL needs to be considered. Our results, however, clearly showed that the Valsalva maneuver does not influence the measured peripapillary vessel density values with the Optovue OCTA system. This result does not mean that breath holding has no influence on ocular blood flow, but it clearly shows that with the SSADA software, slowing the movement of red blood cells does not influence the measured vessel density as long as the red blood cells keep moving in the capillaries of the peripapillary RNFL.

## Future of Optical Coherence Tomography Angiography in the Management of Glaucoma

OCTA appeared in clinical glaucoma research and practice four years ago, and software development is rapid. Results achieved with one software or hardware version may not be valid for the newer versions, which are usually superior to the previous ones. Thus, currently no final decision can be made on the various OCTA parameters’ usefulness for the measurement of glaucomatous progression or conversion of ocular hypertension to glaucoma, their diagnostic value in preperimetric glaucoma, or their utility as indicators of visual field preservation after intraocular pressure-lowering interventions. These limitations, however, are temporary. In the coming years, OCTA will remain one of the most exciting clinical research areas, which deserves attention from both glaucoma specialists and general ophthalmologists treating glaucoma patients.
